# Surface Durability and Mechanical Performance of Sustainable KOH-Activated Hybrid Fly Ash Mortars for Flooring Layers

**DOI:** 10.3390/ma19153216

**Published:** 2026-07-28

**Authors:** Robinson Rúa-Patiño, Edison A. Hincapie-Atehortua, Sergio A. Arboleda-Lopez, Andres F. Urrego-Higuita, M. A. Rico, Ary A. Hoyos-Montilla

**Affiliations:** 1 Faculty of Architecture and Engineering, Institución Universitaria Colegio Mayor de Antioquia, Medellin 050035, Colombia; ehincapi@unal.edu.co (E.A.H.-A.); sergio.arboleda@colmayor.edu.co (S.A.A.-L.); andres.urrego@colmayor.edu.co (A.F.U.-H.); maria.rico@colmayor.edu.co (M.A.R.); 2 Faculty of Engineering, Corporación Universitaria Remington, Medellin 050010, Colombia; 3 Faculty of Mines, Universidad Nacional de Colombia, Medellin 050034, Colombia; 4 Faculty of Architecture, Universidad Nacional de Colombia, Medellin 050034, Colombia; aahoyosm@unal.edu.co

**Keywords:** fly ash, hybrid mortars, alkaline activation, potassium hydroxide, sustainable flooring, surface durability

## Abstract

Sustainable mortars for flooring require mechanical stability and surface durability against abrasion, not only compressive strength. This study evaluates potassium hydroxide (KOH)-activated hybrid fly ash mortars as candidate materials for sustainable flooring and surface wear layers. Ordinary Portland cement (OPC) was partially replaced with fly ash (FA) at OPC/FA ratios of 90/10, 80/20, and 70/30, using 4 M and 8 M KOH solutions. The experimental program included the characterization of fly ash, the alkaline solution, and fine aggregate, as well as flowability, bulk density, compressive strength, abrasion mass loss, and numerical consistency analysis. The results showed that OPC exhibited the highest strength and lowest wear; among the hybrid mortars, 90/10–8 M exhibited the highest relative performance, while 80/20–8 M provided the best balance between cement reduction, strength, and wear. The integrated mechanical–surface performance index $I_{\mathrm{MS}}$ and the analytical–numerical consistency assessment enabled the formulations to be ranked using an integrated selection criterion.

## 1. Introduction

Floors, toppings, and surface wear layers constitute a critical application area within cementitious materials because they are directly exposed to traffic, friction, localized impact, abrasion, and progressive material loss during service. In these elements, compressive strength is necessary but not sufficient: surface quality, curing, workability, compactness, and wear resistance govern the functional durability of the system. In this regard, ACI 302.1R-15 establishes that the performance of concrete floors depends on material strength, placement, finishing, and curing [1]. Complementarily, EN 13892-3:2014 defines procedures for evaluating wear in materials for floor layers [2], whereas ASTM C944/C944M-19 and ASTM C779/C779M-19 enable the abrasion of concrete or mortar surfaces to be quantified under controlled conditions [3,4]. This regulatory framework demonstrates that a mortar intended for sustainable flooring must exhibit both mechanical performance and surface resistance, rather than merely initial load-bearing capacity.

The technical problem is linked to the growing environmental pressure on cementitious materials. In 2004, Gartner argued that reducing emissions from the cement industry requires decreasing clinker content and utilizing industrial by-products with reactive potential [5]. In 2018, Scrivener, John, and Gartner expanded this discussion by noting that eco-efficient cements must combine a low carbon footprint, raw-material availability, and verifiable in-service performance [6]. More recently, the development of codes for low-carbon concrete, such as ACI CODE-323-24, demonstrates that sustainability is no longer solely an environmental concern but now requires a technical basis for specifying and controlling the performance of new cementitious materials [7]. However, this regulatory progress does not by itself resolve the challenges associated with specific applications, such as hybrid mortars for flooring, in which strength, workability, and surface wear must be evaluated in an integrated manner.

Among the approaches for reducing clinker content, fly ash has been one of the most extensively studied precursors. In 1999, Palomo, Grutzeck, and Blanco demonstrated that alkali-activated fly ashes could form cementitious matrices with the potential to partially replace Portland cement [8]. In 2005, Fernández-Jiménez and Palomo showed that the type of alkaline activator modifies the composition and microstructure of fly ash-based systems [9]; in the same year, Bakharev demonstrated that thermal curing accelerates strength development in fly ash geopolymer materials [10]. These studies established the reactivity of fly ash and demonstrated that alkaline activation is sensitive to both the activator and the curing regime; nevertheless, their primary emphasis was on binder formation and overall strength rather than on the surface behavior of mortars intended for flooring or wear layers.

Subsequent research helped explain why formulating an alkali-activated mixture and measuring only its strength are insufficient. In 2006, Mehta and Monteiro explained that the strength and durability of cementitious materials depend on porosity, hydration, and the internal structure of the matrix [11]. In 2006, Shi, Krivenko, and Roy systematized the existing knowledge on alkali-activated cements, highlighting the simultaneous influence of the precursor, activator, and curing conditions [12]. In 2007, Duxson et al. noted that geopolymers are highly sensitive to the chemical composition of the system [13], and in 2010, Ahmaruzzaman showed that fly ash can be valorized in multiple engineering applications [14]. A critical examination of these contributions indicates that the mechanical strength of an activated matrix depends on its chemistry and microstructure; however, sustainable flooring applications additionally require verification that the matrix can produce a compact, placeable, and wear-resistant surface.

At this point, hybrid cements offer an alternative that is more closely aligned with construction applications because they combine Portland cement, fly ash, and alkaline activation. In 2013, García-Lodeiro, Fernández-Jiménez, and Palomo demonstrated that alkali-activated mixtures of fly ash and Portland cement can generate combined reaction products and a progressively more stable matrix [15]. More recently, in 2024, Ahmad et al. analyzed the hydration kinetics and microstructural evolution of high-volume fly ash cements activated with alkaline hydroxides, providing current evidence on the interaction among the Portland cement fraction, fly ash, and alkaline activation [16]. In 2016, García-Lodeiro et al. proposed a descriptive model for hybrid cements with a high fly ash content, emphasizing the coexistence of hydration and alkaline activation products [17]. These studies are relevant because a floor layer requires early strength, dimensional stability, and feasible curing conditions. Nevertheless, this knowledge must still be translated into specific experimental criteria for hybrid fly ash mortars intended for surface wear applications, considering not only the ordinary Portland cement/fly ash (OPC/FA) ratio but also the type and concentration of the selected activator.

In the present study, this discussion focuses on the use of potassium hydroxide (KOH) as an alkaline activator in hybrid OPC/FA matrices. This approach enables the effect of potassium-based activation to be evaluated in mortars with partial replacement of Portland cement by fly ash, considering two experimental KOH concentrations, 4 M and 8 M. The comparison between these alkalinity levels seeks to establish how the activator concentration, together with the OPC/FA ratio, affects compressive strength and surface abrasion; flowability and bulk density are used as control variables for mixture placeability and physical consistency. This approach is supported by studies that have examined the microstructural response of fly ash systems incorporating KOH as part of the activator and the influence of fly ash and activator characteristics on the performance of geopolymer mortars [18,19].

The need to connect microstructure and performance has gained increasing importance in recent years. In 2018, Provis established alkali-activated materials as a recognized family of alternative binders while also noting that their performance depends on precursor chemistry and reaction conditions [20]. In 2026, Rúa-Patiño et al. proposed a chemical–mechanical bridge for alkali-activated concretes, demonstrating that variables such as gel density and interfacial porosity can be translated into measurable mechanical parameters [21]. Although that study focused on anchors rather than flooring, its contribution is relevant because it reinforces a central concept: the performance of an alkali-activated matrix should not be inferred solely from its composition but from how mixture proportioning, the activator, curing, and microstructure translate into mechanical and functional responses.

Based on this background, the research problem lies in the lack of integrated experimental criteria and interpretation tools for evaluating the mechanical–surface performance of KOH-activated hybrid fly ash mortars as candidate materials for sustainable flooring and wear layers. Although the literature demonstrates the reactive capacity of fly ash and the strength development of alkali-activated systems, it remains insufficiently clear how the OPC/FA ratio and KOH concentration jointly affect compressive strength and surface abrasion under controlled preparation and curing conditions. It is hypothesized that, under the adopted curing regime, the OPC/FA ratio and the experimental KOH concentration systematically affect compressive strength and wear response, thereby enabling the identification of hybrid formulations with an improved mechanical–surface balance. Flowability and bulk density are used as control variables for specimen placeability and physical consistency. Within this framework, the objective is to evaluate the mechanical–surface performance of KOH-activated hybrid OPC/FA mortars through compressive strength and abrasion testing, using flowability and density as experimental controls and complementing the analysis with a numerical consistency and sensitivity model. The specific advancement of this work consists of integrating compressive strength and surface abrasion within the same OPC/FA–KOH experimental matrix designed for flooring applications by means of a mechanical–surface comparative criterion, complemented by a parametric consistency and sensitivity analysis. This approach seeks to identify the formulations that provide the best balance among OPC reduction, mechanical performance, and resistance to surface wear.

## 2. Materials and Methods

### 2.1. Materials and Experimental Design

The experimental program was designed to evaluate the mechanical performance and surface durability of KOH-activated hybrid fly ash mortars intended for sustainable flooring and surface wear layer applications. The materials used were OPC, FA, fine aggregate, water, and an alkaline KOH solution. In this study, KOH was used as the alkaline medium for the fly ash fraction at two experimental concentrations, 4M and 8M. The purpose was to evaluate how the alkaline concentration and OPC/FA ratio affect the fresh-state, mechanical, and surface responses of the mortar, without attributing the performance to a single chemical mechanism. The methodology was organized into five stages: (i) material characterization, (ii) mixture design, (iii) specimen preparation and curing, (iv) fresh- and hardened-state evaluation, and (v) surface wear measurement.

Fly ash was considered a mineral precursor within the framework of ASTM C618, which specifies coal fly ash and natural pozzolans for use in cementitious systems [22]. The fine aggregate was characterized before mixture production to reduce the variability associated with the granular phase of the mortar. The characterization included the evaluation of organic impurities in accordance with ASTM C40/C40M [23], the content of material finer than 75 μm by washing in accordance with ASTM C117 [24], particle-size distribution in accordance with ASTM C136/C136M [25], and the relative density and absorption of the fine aggregate in accordance with ASTM C128 [26]. These tests enabled the initial condition of the aggregate to be defined, the effective mixing water to be corrected, and its influence on flowability, compactness, compressive strength, and surface wear to be controlled. Table 1 summarizes the ASTM framework used for characterization and experimental control.

According to the characterization of the fine aggregate, the material corresponded to a natural sand for mortar containing both fine and coarse fractions. The content of material finer than 75 μm was 5.84%, the fineness modulus was 2.88, and the absorption adopted for the initial mixture proportions was 2.04%. These parameters were incorporated into the calculation of effective water, preventing variations in aggregate moisture and absorption from altering the workability, bulk density, mechanical strength, and surface behavior of the mixtures.

Once the aggregate condition and the regulatory control framework had been defined, the experimental matrix of the study was established. This matrix was formulated as a comparative factorial design for the hybrid mixtures, considering three OPC/FA ratios, 90/10, 80/20, and 70/30, and two KOH concentrations, 4M and 8M. The 100% OPC mixture was maintained as the reference without alkaline activation. The total binder content, amount of fine aggregate, and total liquid content were maintained constant in all formulations, whereas the OPC/FA distribution and KOH concentration were varied. Table 2 presents the complete proportions per unit volume used in the experimental program.

The matrix presented in Table 2 enables the effects of the OPC/FA ratio and KOH concentration to be compared within the domain of the activated formulations. The OPC 100/0 mixture serves as the mechanical and surface reference without alkaline activation; however, because it contains no fly ash, it does not constitute a hybrid OPC/FA control without KOH. Consequently, the design does not allow the effect of the activator to be completely isolated from the potential pozzolanic contribution of the fly ash. The scope of the comparison is therefore limited to establishing the effect of changing the concentration from 4M to 8M within each OPC/FA ratio and comparing the activated hybrid formulations with the conventional Portland cement reference.

**Figure 1:**
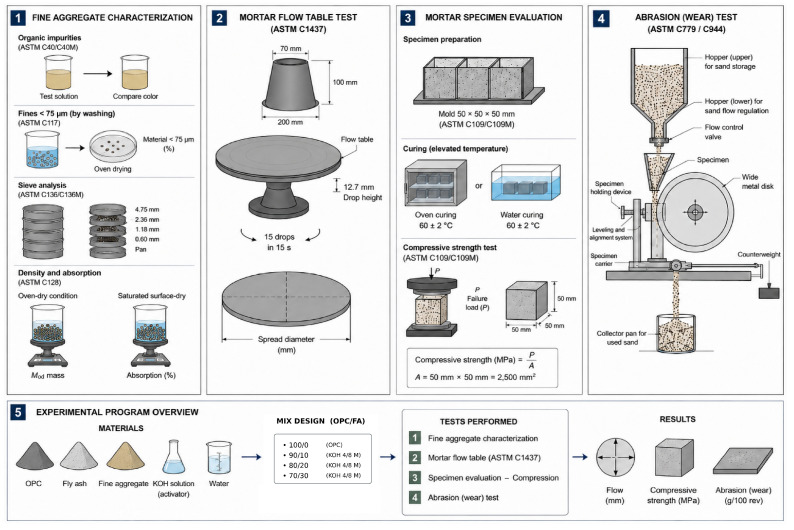
Experimental workflow for KOH-activated hybrid mortars.

Based on the material characterization and the previously defined mixture matrix, Figure 1 presents the operational workflow of the experimental program. The diagram organizes aggregate characterization, flowability control, specimen preparation, compression testing, and surface abrasion evaluation. This structure enables each mixture to be related to the response variables used to assess its performance as a candidate material for sustainable flooring.

Based on the workflow shown in Figure 1, the experimental design was developed as a comparative matrix comprising one 100% OPC reference mixture and six KOH-activated hybrid OPC/FA formulations. The 90/10, 80/20, and 70/30 ratios represented increasing levels of Portland cement replacement with fly ash, whereas the KOH concentrations of 4M and 8M enabled the effect of alkalinity to be evaluated within each replacement level. The 90/10 ratio corresponded to the low level of OPC replacement with FA, whereas the 80/20 and 70/30 ratios extended the comparison to higher fly ash contents. The mixtures were subsequently subjected to the assisted thermal-curing regime at 60 °C defined for the evaluated conditions to promote early strength development and verify the mechanical and surface responses of the hybrid mortars.

For the preparation of the hybrid mortars, the mixing process was organized into two fractions. The first consisted of an OPC–sand paste prepared with the water associated with the Portland cement fraction, whereas the second consisted of an FA–KOH paste prepared with the alkaline solution assigned to the fly ash fraction. Both fractions were subsequently mixed until a homogeneous mortar was obtained. This procedure enabled more direct control of the initial contact between the fly ash and the alkaline activator, improved material placeability, and maintained consistent preparation for the OPC/FA ratios of 90/10, 80/20, and 70/30 evaluated with KOH at 4M and 8M. The reference OPC mixture was prepared with mixing water and without an alkaline solution.

Flowability was evaluated using a flow table in accordance with ASTM C1437 [27], exclusively as an operational control of workability before specimen casting. Four spread diameters $D_{j}$ were recorded for each mixture, and the mean diameter $D_{f}$ was calculated. Because this measurement was used to verify placeability rather than as a response variable of the design, it was not incorporated into the ranking or the mechanical–surface performance comparisons. Compressive strength was determined from the maximum failure load $P_{\max,i}$, recorded in kN, and the loaded area $A_{i}$, expressed in mm^2^, such that the individual strength $f_{m,i}$ was reported in MPa using the conversion factor $10^{3}$. Finally, the mean strength ${\bar{f}}_{m}$ was obtained from the *n* valid specimens for each mixture and testing age:

(1) $$D_{f}=\frac{1}{4}\sum_{j=1}^{4}D_{j}$$

(2) $$f_{m,i}=\frac{10^{3}P_{\max,i}}{A_{i}}$$

(3) $${\bar{f}}_{m}=\frac{1}{n}\sum_{i=1}^{n}f_{m,i}$$

Equations (1)–(3) enabled the initial placeability of the mortar to be controlled, the individual strength of each specimen to be calculated, and the mean mechanical development of the OPC reference mixture to be compared with that of the KOH-activated hybrid mixtures. ASTM C109/C109M [28] was used as a reference during the stages involving 50 mm cubes; subsequently, because of the oxidizing effect of the alkaline solution on the metal molds, PVC cylinders measuring 50 mm in diameter and 100 mm in height were used, while maintaining the same calculation criterion based on maximum load and loaded area.

Bulk density and the surface wear response were calculated from physical variables measured directly in the hardened specimens. Density $\rho_{i}$ was obtained from the mass $m_{i}$ and geometric volume $V_{i}$, determined from the measured dimensions of the cubes or cylinders according to the experimental stage. For the surface abrasion test, conducted in accordance with ASTM C944/C944M, the initial dry mass $m_{0}$, final mass $m_{f}$ after abrasive action, and surface area subjected to wear $A_{a}$ were recorded. These quantities were used to calculate the mass loss Δm and specific abrasion index $I_{a}$:

(4) $$\rho_{i}=\frac{m_{i}}{V_{i}}$$

(5) $$\Delta m=m_{0}-m_{f}$$

(6) $$I_{a}=\frac{\Delta m}{A_{a}}$$

Equations (4)–(6) were used to relate the apparent compactness of the specimens to their material loss under abrasion. In this context, $\rho_{i}$ was interpreted as an auxiliary indicator of compactness, whereas lower values of Δm or $I_{a}$ indicated greater resistance to surface wear. This interpretation is necessary because, in mortars intended for flooring and wear layers, compressive strength alone is insufficient to define the functional performance of the material.

Normalized indicators of mechanical and surface performance were defined to compare the hybrid mixtures with the OPC reference mixture. The relative mechanical index $I_{m}\left(t\right)$ relates the mean strength of the hybrid mixture ${\bar{f}}_{m,\mathrm{hyb}}\left(t\right)$ to the mean strength of the OPC mixture ${\bar{f}}_{m,\mathrm{OPC}}\left(t\right)$, evaluated at the same age *t*. Complementarily, the normalized abrasion index $I_{a,\mathrm{norm}}$ compares the specific abrasion index of the evaluated mixture $I_{a,\mathrm{mix}}$ with that of the reference mixture $I_{a,\mathrm{OPC}}$. Finally, the integrated mechanical–surface performance index $I_{\mathrm{MS}}$ relates the 28-day mechanical response to the normalized wear response:

(7) $$I_{m}\left(t\right)=\frac{{\bar{f}}_{m,\mathrm{hyb}}\left(t\right)}{{\bar{f}}_{m,\mathrm{OPC}}\left(t\right)}$$

(8) $$I_{a,\mathrm{norm}}=\frac{I_{a,\mathrm{mix}}}{I_{a,\mathrm{OPC}}}$$

(9) $$I_{\mathrm{MS}}=\frac{I_{m}\left(28\right)}{I_{a,\mathrm{norm}}}$$

Equations (7)–(9) enabled the relative strength, normalized wear, and mechanical–surface balance of the evaluated mixtures to be compared. Values of $I_{m}\left(t\right)$ close to unity indicate mechanical performance comparable to that of OPC, whereas values of $I_{a,\mathrm{norm}}>1$ reflect greater relative wear than the reference mixture. For OPC, $I_{\mathrm{MS}}=1.000$ because both strength and abrasion are normalized with respect to this reference. In the hybrid mixtures, higher values of $I_{\mathrm{MS}}$ indicate a better compromise between compressive strength and lower susceptibility to surface wear. $I_{\mathrm{MS}}$ is a dimensionless comparative index constructed from macroscopic performance variables and does not constitute a constitutive material property.

The quantitative treatment was conducted on a descriptive basis using the mean values obtained for each formulation. The correlation coefficients *r* and $R^{2}$ were used to describe associations among the values aggregated by mixture and not as hypothesis tests or evidence of statistical significance. Because confidence intervals were not estimated and inferential tests were not applied, the observed differences were interpreted exclusively within the evaluated experimental dataset and not as statistically significant or generalizable differences.

### 2.2. Numerical Consistency and Sensitivity Model

The numerical model was formulated as a parametric consistency and sensitivity tool for interpreting the experimental trends of the KOH-activated hybrid mortars. Its purpose was not to predict failure or provide independent validation of the tests, but rather to evaluate how relative changes in initial stiffness affect the normalized elastic response and whether this response preserves the experimentally observed performance ranking. The formulation was restricted to the linear–elastic, small-strain regime; therefore, it does not represent cracking, damage, plasticity, or post-peak behavior. The practical contribution of the model consists of providing a dimensionless and reproducible framework for organizing the formulations, identifying mechanically inconsistent trends, and prioritizing mixtures for more complex experimental or numerical evaluations.

The computational domain was defined from the nominal geometry of the cylindrical specimens used in the comparative compression stages. A three-dimensional domain Ω was considered, described by the spatial coordinates *x*, *y*, and *z*, the mean specimen diameter *D*, and the mean specimen height *H*. The cross-section was represented as a circular region with a radius of D/2, whereas the height was bounded between the base and the top face of the cylinder:

(10) $$\Omega =\left\{\left(x,y,z\right)\in R^{3}:x^{2}+y^{2}\leq {\left(\frac{D}{2}\right)}^{2},\;0\leq z\leq H\right\}.$$

Equation (10) reproduces the geometric condition of the PVC cylinders measuring 50 mm in diameter and 100 mm in height used in compression. The bottom base was constrained in the vertical direction, the top face was subjected to a prescribed axial displacement, and the lateral surface was left traction-free to represent a condition without lateral confinement. Additionally, minimum constraints were imposed at reference points on the base to eliminate rigid-body modes without artificially modifying the mechanical response of the specimen.

Under these boundary conditions, specimen equilibrium was formulated in weak form. A displacement field $u\in {\left[H^{1}\left(\Omega \right)\right]}^{3}$, compatible with the imposed constraints, was sought such that the virtual internal work was zero for every admissible test field v. In this expression, σ(u) represents the stress tensor associated with the displacement, ε(v) is the virtual strain tensor, and dΩ is the differential volume:

(11) $$\int_{\Omega }\sigma \left(u\right):\varepsilon \left(v\right)\;d\Omega =0.$$

Equation (11) expresses internal equilibrium under an imposed displacement; therefore, the compressive load is not prescribed directly as an external traction but is subsequently obtained as the reaction on the loaded face. This strategy is consistent with the experimental test, in which the applied axial displacement generates a load response dependent on the initial stiffness of each mixture.

The constitutive relationship was assumed to be linear elastic and isotropic. In this case, the Cauchy stress tensor σ was related to the infinitesimal strain tensor ε through the constitutive tensor C(E,ν), defined by the elastic modulus *E* and Poisson’s ratio ν. Because direct measurements of *E* were not available for all mixtures, this parameter was treated as a variable normalized with respect to OPC:

(12) σ=C(E,ν):ε.

Because direct measurements of the elastic modulus were not available for all mixtures, *E* was treated as a parametric variable normalized with respect to OPC through $\chi_{E}$. Consequently, the model does not identify the actual elastic modulus of each formulation but quantifies the sensitivity of the initial response to relative changes in stiffness. This parameterization enables the mechanical consistency of the experimental ranking to be examined without attributing independent predictive capability to the model.

Two main quantities were obtained from the elastic solution: the numerical reaction force and the normalized initial stiffness. The reaction $P_{\mathrm{num}}$ was calculated by integrating the normal stress over the loaded top boundary $\Gamma_{t}$, where n is the outward normal vector, $e_{z}$ is the unit vector in the axial direction, and dΓ is the differential surface area. The initial stiffness *K* was obtained from the slope of the initial segment of the load–displacement curve and was normalized as $K_{n}$, using the slope corresponding to OPC as the reference:

(13) $$P_{\mathrm{num}}=\left|\int_{\Gamma_{t}}\sigma n\cdot e_{z}\;d\Gamma \right|$$

(14) $$K_{n}=\frac{K_{\mathrm{hyb}}}{K_{\mathrm{OPC}}}$$

Equations (13) and (14) enable the reaction force to be obtained and the initial stiffness of the parametric scenarios to be compared with that of OPC. Although $K_{n}$ is calculated from the initial slope of the numerical load–displacement curve, its value depends directly on the normalized stiffness level assigned through $\chi_{E}$. Therefore, $K_{n}$ does not constitute an independent measurement and is not used to validate $I_{\mathrm{MS}}$; its comparison with the experimental index is limited to evaluating internal consistency and preservation of the ranking among formulations.

Mesh independence was verified through successive refinements of the characteristic finite-element size. For each refinement level, the relative variation $\Delta Q_{h}$ of a control quantity $Q_{h}$, taken as the initial stiffness *K* or reaction force $P_{\mathrm{num}}$, was calculated. The terms $Q_{h_{i}}$ and $Q_{h_{i-1}}$ represent the values obtained using two consecutive meshes, and $h_{i}$ corresponds to the characteristic element size at the evaluated refinement level:

(15) $$\Delta Q_{h}=\left|\frac{Q_{h_{i}}-Q_{h_{i-1}}}{Q_{h_{i}}}\right|\times 100.$$

Equation (15) enabled changes associated with numerical refinement to be distinguished from those attributable to material behavior. The solution was considered mesh-independent when $\Delta Q_{h}<3\%$, a criterion adopted to prevent purely discretization-related variations from being interpreted as actual differences among mixtures.

The sensitivity of the model was organized using dimensionless parameters linked to the experimental results. The stiffness factor $\chi_{E}$ relates the modulus assigned to the hybrid mixture to the OPC reference modulus; the strength factor $\chi_{f}\left(t\right)$ was taken as equal to the relative mechanical index previously defined experimentally; and the compactness factor $\chi_{\rho }$ compares the mean bulk density of the hybrid mixture with the mean bulk density of the reference mixture. The previously defined expressions for ${\bar{f}}_{m}$ and bulk density are retained in this section to avoid redundancy:

(16) $$\chi_{E}=\frac{E_{\mathrm{hyb}}}{E_{\mathrm{OPC}}}$$

(17) $$\chi_{f}\left(t\right)=I_{m}\left(t\right)=\frac{{\bar{f}}_{m,\mathrm{hyb}}\left(t\right)}{{\bar{f}}_{m,\mathrm{OPC}}\left(t\right)}$$

(18) $$\chi_{\rho }=\frac{{\bar{\rho }}_{\mathrm{hyb}}}{{\bar{\rho }}_{\mathrm{OPC}}}$$

Equations (16)–(18) organize the comparison between parametric stiffness and the experimental strength and compactness indicators. Only $\chi_{E}$ modifies the constitutive tensor of the FEM model; $\chi_{f}\left(t\right)$ and $\chi_{\rho }$ are subsequently used as external comparison variables and not as constitutive properties or evidence of independent validation.

The abrasion response was incorporated through the relative wear factor $\chi_{w}$, equivalent to the normalized abrasion index defined previously. In this relationship, $I_{a,\mathrm{hyb}}$ corresponds to the specific abrasion index of the hybrid mixture, and $I_{a,\mathrm{OPC}}$ corresponds to the specific abrasion index of the reference mixture. In addition, alkaline activation was classified using the vector $p_{\mathrm{act}}$, composed of the molarity of the KOH solution, $M_{\mathrm{KOH}}$, and the mass ratio between Portland cement and fly ash, $r_{\mathrm{OPC}/\mathrm{FA}}$:

(19) $$\chi_{w}=\frac{I_{a,\mathrm{hyb}}}{I_{a,\mathrm{OPC}}}$$

(20) $$p_{\mathrm{act}}={\left[M_{\mathrm{KOH}},r_{\text{OPC/FA}}\right]}^{T}$$

Equation (19) defines an experimental indicator of relative wear, where values lower than unity indicate less material loss relative to OPC. $\chi_{w}$ does not enter the constitutive equation or the solution of the FEM problem but is used in the subsequent comparison between the numerical response and surface performance. Equation (20) does not act as a constitutive law but as a classification variable for organizing the mixtures according to their alkaline concentration and OPC/FA proportion, thereby enabling the formulation parameters to be related to the mechanical and surface trends.

The computational implementation was performed in a finite-element environment such as FEniCS, with geometry and mesh generation in Gmsh, followed by the solution of the variational problem [29,30,31]. The direct FEM inputs were the specimen geometry, prescribed displacement, Poisson’s ratio, and parametric stiffness level defined through *E* or $\chi_{E}$. The numerical outputs were $P_{\mathrm{num}}$ and $K_{n}$. Strength, density, abrasion, and $I_{\mathrm{MS}}$ remained external experimental indicators used exclusively to evaluate the consistency of the resulting ranking.

**Figure 2:**
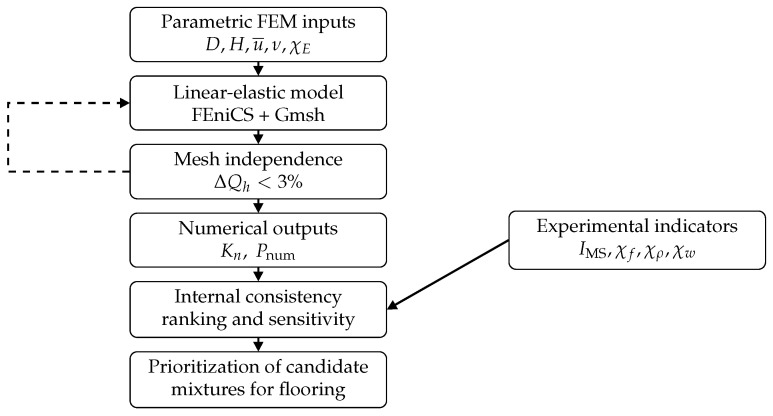
Parametric workflow of the FEM model and comparison with the experimental indicators.

Figure 2 separates the parametric FEM workflow from the experimentally obtained indicators. The two branches converge only in the consistency assessment, thereby avoiding the interpretation of the experimental indicators as constitutive inputs or the numerical response as independent validation. This organization enables the model to be used as a tool for sensitivity analysis, interpretation, and formulation prioritization.

## 3. Results

### 3.1. Material Characterization

Material characterization enables the relationship between the physical and chemical nature of the components and the expected performance of KOH-activated hybrid mortars to be established. In this study, fly ash constitutes the reactive precursor, the KOH solution controls the level of alkaline activation, and the fine aggregate influences the workability, compactness, and surface response of the mortar. Therefore, before discussing compressive strength and wear, it is necessary to verify whether the materials used exhibit characteristics compatible with a hybrid cementitious matrix intended for flooring and surface wear layers.

**Figure 3:**
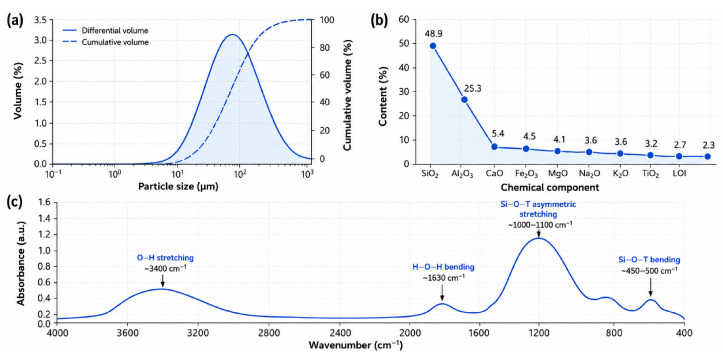
Physical and chemical characterization of the fly ash.

Figure 3 summarizes the physical and chemical characterization of the fly ash used as a precursor in the hybrid mixtures. In panel (Figure 3a), the particle-size distribution shows a dominant fraction within the micrometric range, with a well-defined differential curve and a continuous cumulative curve, suggesting a particle distribution suitable for contributing to the packing of the cementitious matrix. This condition is relevant for mortars intended for surface layers because a well-distributed fine phase can promote initial compactness and reduce internal voids associated with strength loss or wear. In panel (Figure 3b), the chemical composition shows a predominance of ${\mathrm{SiO}}_{2}$ and ${\mathrm{Al}}_{2}O_{3}$, with contents of 48.9% and 25.3%, respectively, whereas CaO, ${\mathrm{Fe}}_{2}O_{3}$, MgO, ${\mathrm{Na}}_{2}O$, $K_{2}O$, ${\mathrm{TiO}}_{2}$, and loss on ignition are present in lower proportions. This composition confirms the silicoaluminous nature of the fly ash and supports its selection as a precursor compatible with alkaline activation within an OPC/FA matrix. Finally, panel (Figure 3c) identifies bands associated with O–H stretching, H–O–H bending, and Si–O–T vibrations, where *T* represents Si or Al, which is consistent with aluminosilicate networks reported for fly ashes [32]. Overall, the results shown in Figure 3 indicate that the fly ash exhibits a favorable combination of fineness, silicoaluminous composition, and spectroscopic response for evaluating its potential contribution to the performance of KOH-activated hybrid mortars.

**Figure 4:**
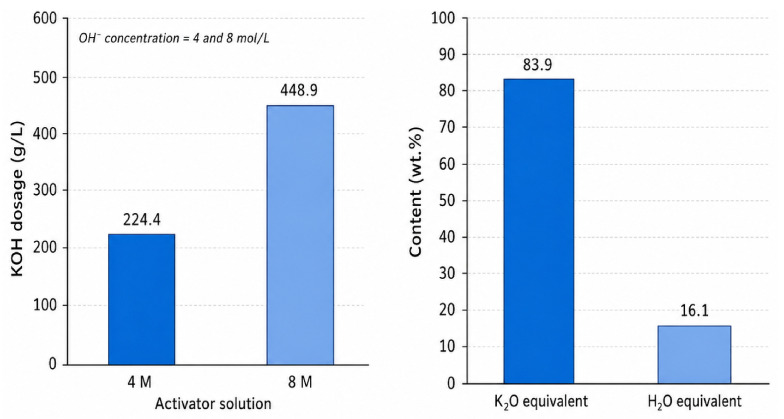
Experimental concentrations and equivalent composition of the KOH solution.

Figure 4 presents the two activation levels used in the experimental program and the equivalent expression of KOH. The 4 M and 8 M solutions contain 224.4 g/L and 448.9 g/L of KOH, respectively, and represent an increase in the nominal concentration of ${\mathrm{OH}}^{-}$ ions. From a chemical perspective, greater availability of ${\mathrm{OH}}^{-}$ may promote the cleavage and dissolution of Si–O–Si and Si–O–Al bonds in the reactive fraction of the fly ash, whereas $K^{+}$ ions participate in charge compensation and may modify the cross-linking of aluminosilicate products [33]. In a hybrid OPC/FA matrix, these products may coexist and interact with calcium-rich hydrates originating from the Portland cement fraction; therefore, the response should not be attributed to a single type of gel [34]. However, an increase in molarity does not necessarily correspond to a linear increase in the degree of reaction because it also modifies solution viscosity, ionic mobility, water availability, precipitation rate, and workability. In the right panel, KOH is expressed in stoichiometric terms of equivalent $K_{2}O$ and equivalent water. In this study, the comparison between 4 M and 8 M is used to identify macroscopic performance differences, but not to demonstrate the formation of specific phases or reaction products.

**Figure 5:**
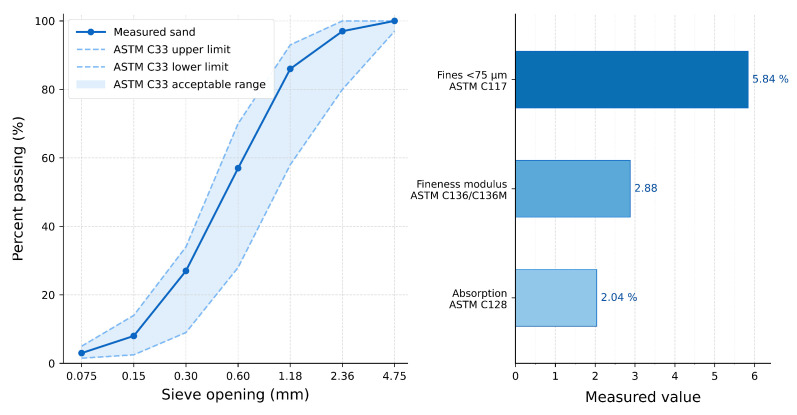
Particle-size and physical characterization of the fine aggregate.

Figure 5 shows the particle-size and physical characterization of the fine aggregate used in the mixtures. In the left panel, the percentage-passing curve of the measured sand is compared with the reference limits of ASTM C33, showing a continuous distribution within the acceptable range for fine aggregates. This condition is important because a continuous particle-size distribution promotes packing of the granular phase and reduces the likelihood of abrupt variations in paste demand, flowability, and mortar compaction. In the right panel, the complementary physical indicators show a content of fines smaller than 75μm of 5.84%, a fineness modulus of 2.88, and an absorption of 2.04%. These values enable three critical design aspects to be controlled: the amount of fines that may modify water demand, the overall gradation that influences workability, and the absorption that must be considered when adjusting the effective mixing water. Overall, the results shown in Figure 5 indicate that the fine aggregate exhibits suitable conditions for use in mortars, preventing the granular phase from introducing excessive variability in flowability, bulk density, mechanical strength, and surface response to wear [35].

Overall, the characterization confirms that the materials used are compatible with the purpose of the study and with the formulation of hybrid mortars for surface layers subjected to wear. The fly ash exhibits a fine fraction and a silicoaluminous composition compatible with alkaline and pozzolanic processes; the KOH solution enables the effect of two alkalinity levels within the OPC/FA matrix to be evaluated; and the fine aggregate exhibits a particle-size distribution, fine content, and absorption that enable workability and effective mixing water to be controlled. This material basis supports the experimental control of placeability and the subsequent evaluation of compressive strength and surface abrasion, which are the variables used to compare the mechanical–surface performance of the KOH-activated OPC/FA mixtures.

### 3.2. Mechanical Performance and Surface Durability

**Figure 6:**
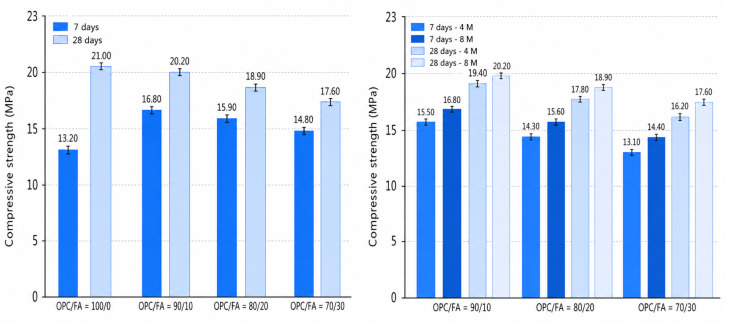
Compressive strength of OPC and OPC/FA hybrid mortars activated with KOH at 4 M and 8 M.

Mechanical evaluation constitutes the first level of performance verification for KOH-activated hybrid mortars because a floor layer must develop a sufficiently strong matrix before being subjected to surface abrasion. In this study, compressive strength is interpreted as an integrated response of the OPC/FA matrix involving the silicoaluminous composition of the fly ash, the alkalinity level provided by KOH, and the particle-size stability of the previously characterized fine aggregate. Figure 6 enables this interaction to be evaluated at two scales: the left panel compares the strength development of the OPC mixture and the 90/10, 80/20, and 70/30 OPC/FA ratios, whereas the right panel separates the 4 M and 8 M conditions for each hybrid ratio. The values shown correspond to the means of the replicate specimens, and the error bars represent the standard deviation obtained for each mixture and testing age. Thus, strength is not analyzed as an isolated value but as an integrated response to the effects of the OPC/FA ratio and KOH concentration on the mechanical performance of the evaluated formulations.

The results indicate that the compressive response of the hybrid mixtures depends jointly on the OPC/FA ratio and KOH concentration. The OPC 100/0 mixture is maintained as the internal reference for the experimental program; therefore, its strength should be interpreted under the adopted mixture proportioning, geometry, preparation, and curing conditions rather than as a nominal characterization of the cement. For each OPC/FA ratio, the comparison between 4M and 8M enables the effect of alkalinity on strength development to be evaluated; in turn, for the same KOH concentration, the comparison among 90/10, 80/20, and 70/30 enables the effect of increasing fly ash content to be analyzed. Overall, the reduction in the Portland cement fraction modifies the final load-bearing capacity. Therefore, the selection of a formulation for floor layers should not be based solely on compressive strength but should also be compared with abrasion mass loss and normalized indicators of surface performance [36].

**Figure 7:**
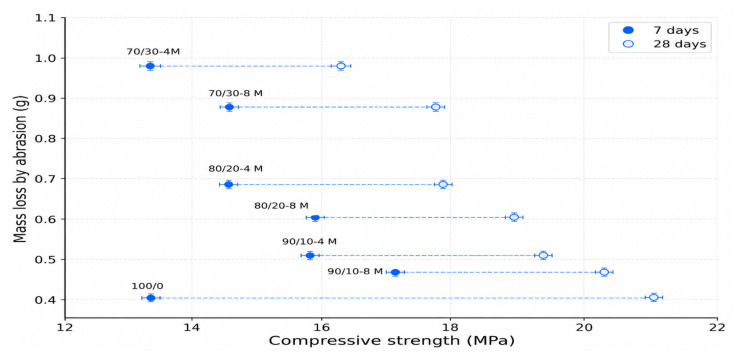
Relationship between compressive strength and abrasion mass loss at 7 and 28 days.

Figure 7 enables the previously discussed mechanical response to be related to the surface durability of the evaluated mortars. In general, mixtures with higher compressive strength tend to be located in the region of lower abrasion mass loss, whereas formulations with lower strength exhibit greater surface material loss. Each point corresponds to the mean value obtained from the replicate specimens; the horizontal error bars represent the standard deviation of compressive strength, and the vertical error bars represent the standard deviation of abrasion mass loss. This trend shows that, under the evaluated conditions, an increase in load-bearing capacity was associated with lower susceptibility to surface material loss. This behavior is consistent with greater matrix cohesion, as reported for cementitious and alkali-activated systems, although this interpretation does not constitute direct microstructural characterization. In this regard, the OPC/FA 100/0 mixture serves as the lower-wear reference, whereas the 90/10, 80/20, and 70/30 formulations enable evaluation of how cement replacement and KOH concentration modify surface stability.

The comparison between 4M and 8M within each OPC/FA ratio enables the effect of activator concentration on abrasion mass loss to be identified. At the same time, the comparison among 90/10, 80/20, and 70/30 enables the effect of increasing fly ash content on surface resistance to be observed. Under this interpretation, the formulation of greatest interest should not be defined solely by the lowest absolute mass loss but by the balance among Portland cement reduction, compressive strength, and wear response. This criterion is particularly relevant for mortars intended for sustainable flooring and surface layers subjected to abrasion [37].

**Figure 8:**
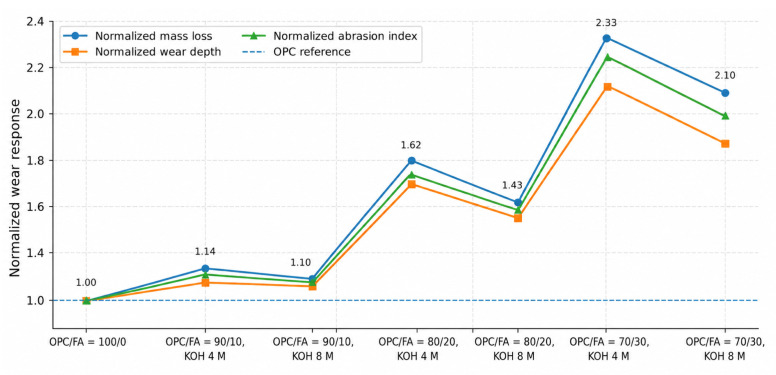
Normalized wear response with respect to the OPC reference mortar.

Figure 8 presents the wear response normalized with respect to the OPC mixture, which is taken as the unit reference. This normalization enables direct comparison of the 90/10, 80/20, and 70/30 hybrid formulations activated with KOH at 4M and 8M. Values greater than unity indicate greater relative material loss compared with the OPC reference, whereas values closer to unity reflect a surface response closer to that of the reference mixture. Therefore, the figure enables the effects of cement replacement and activator molarity on susceptibility to wear to be evaluated simultaneously.

These results maintain the trend observed in Figure 6 and Figure 7: the OPC/FA proportion and KOH concentration jointly modify the mechanical–surface performance of the hybrid mortars. From a phenomenological perspective, a reduction in normalized mass loss with increasing activator concentration would suggest a more cohesive surface matrix; however, the magnitude of this effect must be interpreted within each OPC/FA ratio and not as a trend that can be extrapolated beyond the evaluated conditions. Consequently, the selection of the most suitable mixture should consider strength, normalized wear, and the actual level of Portland cement reduction [38].

**Figure 9:**
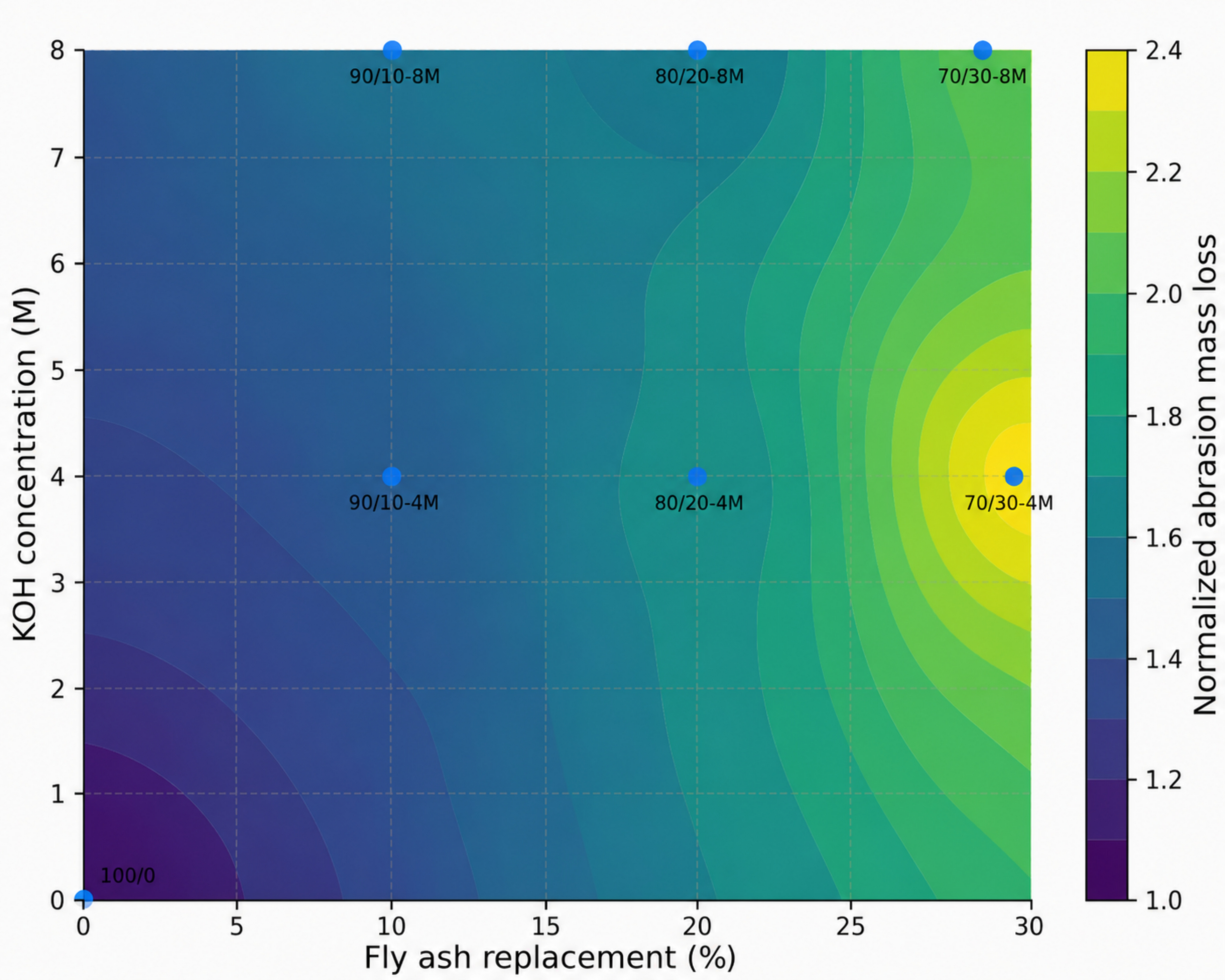
Interpolated surface of normalized abrasion mass loss as a function of fly ash replacement and KOH concentration.

The joint influence of fly ash replacement and KOH concentration is summarized in Figure 9. The interpolated surface was constructed from the 90/10, 80/20, and 70/30 hybrid formulations evaluated with KOH at 4M and 8M. In this analysis, the OPC mixture is maintained as the normalization reference but does not represent a KOH-activated condition. Therefore, the surface should be interpreted as an exploratory visualization of the wear response within the hybrid experimental domain and not as a predictive surface outside the tested levels.

The surface enables observation of how normalized mass loss changes when the fly ash content and activator concentration vary simultaneously. This representation helps identify regions with a better mechanical–surface balance and more critical wear conditions. However, because of the limited number of experimental levels, the surface should not be interpreted as a complete statistical optimization but as a graphical tool for ordering the observed trends. Under this criterion, material sustainability should not be evaluated solely by increasing the fly ash content but through the balance among cement reduction, strength, alkaline activation, and surface durability.

### 3.3. Integrated Mechanical–Surface Performance Evaluation

**Figure 10:**
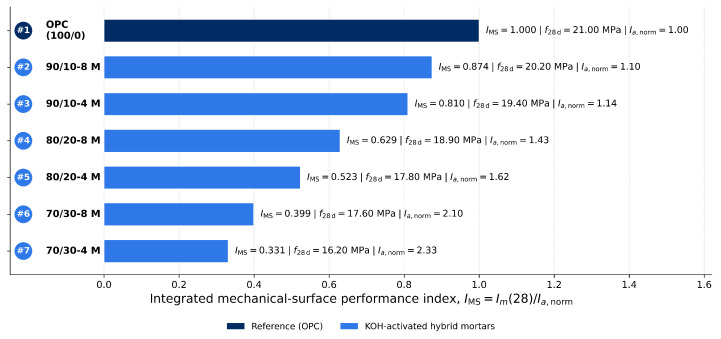
Experimental ranking of the integrated mechanical–surface performance index for OPC and KOH-activated hybrid mortars.

The evaluation of the integrated mechanical–surface performance index was proposed as a consistency assessment between the mechanical strength and surface durability of the evaluated mortars. After analyzing compressive strength, abrasion mass loss, and the normalized wear response, this subsection integrates these results through $I_{\mathrm{MS}}$, defined in Equation (9). Figure 10 presents the experimental ranking of the index for the OPC reference mixture and the 90/10, 80/20, and 70/30 hybrid formulations with KOH at 4M and 8M. OPC is taken as the reference with $I_{\mathrm{MS}}=1.000$, whereas the hybrid mixtures are ranked based on the relationship between the 28-day relative mechanical index and the normalized abrasion response.

The ranking observed in Figure 10 is consistent with the previous compression and wear results. OPC retains the reference value with $I_{\mathrm{MS}}=1.000$, followed by 90/10–8 M and 90/10–4 M, with $I_{\mathrm{MS}}=0.874$ and 0.810, respectively. Among the mixtures with higher replacement levels, 80/20–8 M outperforms 80/20–4 M, whereas 70/30–8 M and 70/30–4 M exhibit the lowest values. Under this criterion, 90/10–8 M exhibits the highest relative performance, whereas selection of the most suitable alternative for sustainable flooring should also consider the percentage reduction in Portland cement.

**Figure 11:**
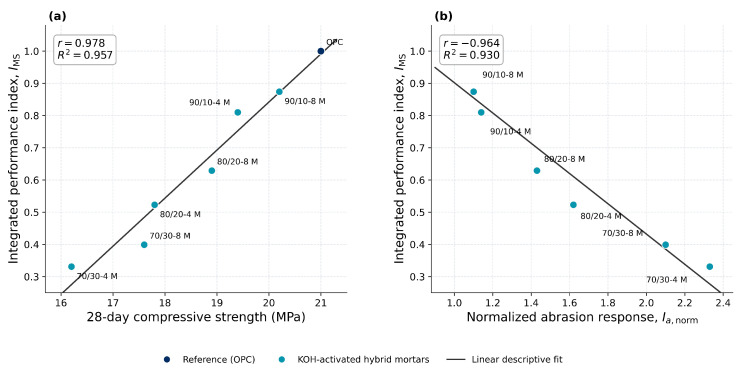
Correlation of the integrated mechanical–surface performance index with (**a**) 28-day compressive strength and (**b**) normalized abrasion response.

The descriptive association of the index with its constituent variables is presented in Figure 11. In Figure 11a, $I_{\mathrm{MS}}$ exhibits a positive correlation with 28-day compressive strength, with r=0.978 and $R^{2}=0.957$. In Figure 11b, the index exhibits a negative correlation with normalized abrasion, with r=−0.964 and $R^{2}=0.930$. Because $I_{\mathrm{MS}}$ is mathematically constructed from relative strength and normalized abrasion, these coefficients describe the internal consistency of the index and do not constitute independent statistical validation or a test of significance among formulations.

Figure 10 shows that 90/10–8 M exhibits the highest performance among the hybrid formulations, with $I_{\mathrm{MS}}=0.874$, and constitutes the preferred alternative when only the mechanical–surface response is prioritized. The 80/20–8 M formulation, with $I_{\mathrm{MS}}=0.629$, a compressive strength of 18.90 MPa, and a normalized abrasion index of 1.43, neither exceeds nor equals its performance. Its technical relevance lies in doubling the replacement of OPC by FA from 10% to 20% while maintaining an intermediate response within the evaluated dataset. Therefore, the recommendation is conditional: 90/10–8 M provides the highest relative performance, whereas 80/20–8 M represents a compromise alternative among OPC replacement, strength, and abrasion.

### 3.4. Analytical–Numerical Consistency Assessment

The analytical–numerical assessment examines whether the normalized elastic response of the model preserves the experimental ranking defined by $I_{\mathrm{MS}}$. The stiffness $K_{n}$ is obtained from the parametric stiffness scenarios, whereas $I_{\mathrm{MS}}$ is constructed from relative strength and normalized abrasion. Because both components are organized within the same experimental framework, their comparison constitutes an internal consistency assessment and not an independent validation or an evaluation of predictive capability.

**Figure 12:**
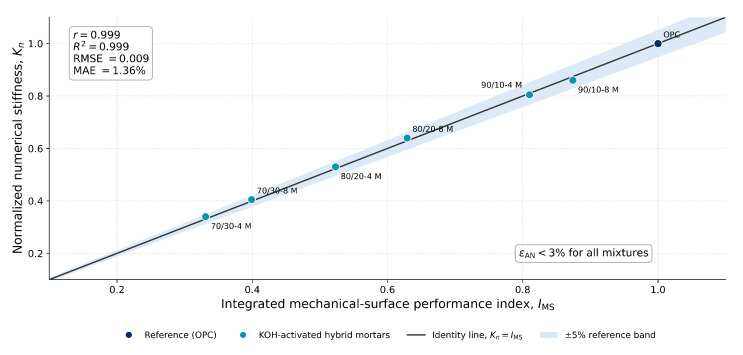
Internal-consistency comparison between the integrated mechanical–surface performance index and normalized numerical stiffness.

Figure 12 presents the comparison between $I_{\mathrm{MS}}$ and $K_{n}$. The identity line and the ±5% reference band enable the proximity between both normalized rankings to be visualized. The values of r=0.999, $R^{2}=0.999$, an RMSE of 0.009, and an MAE of 1.36% describe the internal agreement of the analyzed dataset but do not represent predictive-validation metrics against independent data.

To quantify the proximity between the integrated experimental index and the numerical response, the relative consistency difference $\varepsilon_{\mathrm{AN}}$, calculated from $K_{n}$ and $I_{\mathrm{MS}}$, was used. This quantity describes the agreement between the two rankings and does not represent a predictive error against independent data:

(21) $$\varepsilon_{\mathrm{AN}}=\left|\frac{K_{n}-I_{\mathrm{MS}}}{I_{\mathrm{MS}}}\right|\times 100.$$

**Figure 13:**
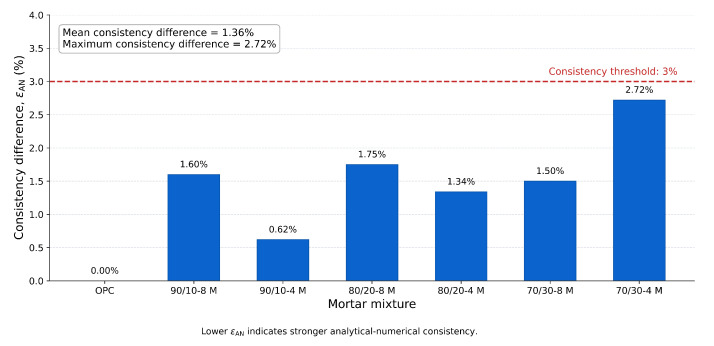
Relative analytical–numerical consistency difference for OPC and KOH-activated hybrid mortars.

In Equation (21), $\varepsilon_{\mathrm{AN}}$ represents the relative consistency difference between the normalized stiffness $K_{n}$ and the experimental index $I_{\mathrm{MS}}$. Figure 13 presents values below 3%, with a mean difference of 1.36% and a maximum difference of 2.72%. These results demonstrate proximity between the rankings within the adopted parametric framework but do not constitute independent validation.

Overall, Figure 12 and Figure 13 show that the parametric model preserves the performance ranking defined by $I_{\mathrm{MS}}$. This result supports its use as a consistency and sensitivity tool, but not as independent validation or as a predictive failure model. Its practical value lies in organizing the formulations and identifying those that require additional experimental characterization or nonlinear simulations.

## 4. Discussion

The decline in mechanical performance with increasing replacement of OPC by FA and the relatively more favorable response of 8 M in some formulations are consistent with studies on hybrid cements, in which strength development depends simultaneously on the Portland cement fraction, fly ash reactivity, activator concentration, and curing regime [15,16,17,19]. Under the adopted conditions, the lower performance of the 70/30 formulation indicates that the increase in FA was not fully compensated by KOH activation; this result is limited to the raw materials, liquid content, and curing conditions employed and does not demonstrate that 30% replacement is unfavorable in other systems. Unlike studies focused primarily on hydration or strength, the present analysis compares the mechanical response with surface wear. From this perspective, 90/10–8 M exhibits the highest relative performance, whereas 80/20–8 M represents a compromise alternative among OPC replacement, strength, and abrasion.

Within this chemical framework, the better performance observed with 8 M in some formulations is consistent with changes in dissolution and reaction kinetics relative to 4 M, but does not constitute evidence of a larger amount of gel or of a specific phase. The response also depends on the OPC/FA ratio, available calcium, water content, and curing regime. The identification and quantification of unreacted fly ash, calcium-rich hydrates, and alkaline aluminosilicate products would require characterization of the hardened mortars by XRD, FTIR, TGA, and SEM–EDS. Consequently, activation chemistry is used here as an interpretive framework supported by the literature and not as an experimentally demonstrated mechanism.

The inverse association observed between compressive strength and abrasion mass loss is qualitatively consistent with studies relating greater mechanical capacity to lower susceptibility to wear in alkali-activated fly ash systems [37]. In the formulations evaluated here, the increase in FA was accompanied by a reduction in strength and an increase in wear, whereas 8 M produced a relatively more favorable response for some OPC/FA ratios. This indicates that the effect of alkaline concentration depends on binder composition and does not constitute a uniform or generalizable improvement. Because an OPC/FA mixture without KOH was not included, the effect of the activator cannot be completely separated from the potential pozzolanic contribution of FA. Furthermore, the absence of characterization of the hardened mortars precludes attributing these trends to specific reaction products or microstructural changes.

Abrasion was adopted as the durability indicator directly related to the surface function considered in this study. However, the experimental program did not include water absorption of the hardened mortar, open porosity, permeability, shrinkage, freeze–thaw resistance, or chemical exposure. Consequently, the results enable preliminary selection based on mechanical performance and surface resistance, but do not establish the overall durability or long-term behavior of the formulations. These properties must be verified before specifying the material under actual service conditions.

The specific contribution of the numerical component does not lie in providing independent validation, but in explicitly separating the FEM parametric variables from the experimental indicators and evaluating the consistency between normalized initial stiffness and mechanical–surface performance. This structure provides a transparent framework for organizing and prioritizing formulations without attributing predictive capability to the elastic model. Its practical value lies in identifying stiffness trends consistent with the experimental ranking, whereas the prediction of cracking, damage, and post-peak response requires measured mechanical properties, nonlinear constitutive models, and independent validation data.

## 5. Conclusions

Based on the results obtained under the evaluated experimental conditions, the main conclusions are as follows:

- Under the evaluated experimental conditions, the observed descriptive trends are consistent with the study hypothesis: the OPC/FA ratio and KOH concentration were associated with variations in compressive strength and surface wear response, enabling the performance of the hybrid formulations to be differentiated.
- Among the hybrid formulations evaluated, the OPC/FA 80/20 condition activated with 8 M KOH was identified as an alternative of considerable technical interest when considering the balance among partial Portland cement reduction, compressive strength, and surface wear response.
- The comparison between 4 M and 8 M KOH enabled the effect of activator concentration within each OPC/FA ratio to be identified, showing that alkalinity simultaneously modifies the mechanical response and susceptibility to surface wear.
- Portland cement replacement of up to 30% increased susceptibility to wear and reduced the final load-bearing capacity; therefore, the 70/30 mixtures do not represent the most favorable option under the activation and curing regime employed.
- The relationship between compressive strength and abrasion mass loss exhibited an inverse trend but confirmed that mechanical strength should not be used as the sole selection criterion for mortars intended for flooring or wear layers.
- The integrated mechanical–surface performance index $I_{\mathrm{MS}}$ related relative strength and normalized abrasion through a single comparison criterion. Its behavior was consistent with the experimental ranking and enabled the functional balance of the evaluated formulations to be compared.
- The analytical–numerical consistency assessment showed that the parametric elastic model preserves the ranking defined by $I_{\mathrm{MS}}$. Its scope is limited to the analysis of initial stiffness and sensitivity; therefore, it does not constitute independent validation or a prediction of the nonlinear response of the mortars.
- The results are applicable to the raw materials, OPC/FA ratios, KOH concentrations, preparation procedure, and curing regime employed. Abrasion represents only the evaluated component of surface durability; therefore, long-term application requires the investigation of water absorption, porosity, permeability, shrinkage, freeze–thaw cycles, chemical durability, additional ages, and more representative service conditions.

## Figures and Tables

**Table 1:** ASTM standards used for characterization and experimental control.

Test	Standard	Use in the Study
Fly ash	ASTM C618	Reference for the use of fly ash as a cementitious precursor.
Organic impurities	ASTM C40/C40M	Control of organic contamination in the fine aggregate.
Fines <75μm	ASTM C117	Determination of material removable by washing.
Particle-size distribution	ASTM C136/C136M	Particle-size distribution and fineness modulus.
Density and absorption	ASTM C128	Correction of the effective mixing water.
Flowability	ASTM C1437	Control of fresh-state workability.
Compression	ASTM C109/C109M	Determination of the mechanical strength of the mortar.
Surface abrasion	ASTM C944/C944M	Evaluation of surface wear in candidate mortars for flooring.

**Table 2:** Experimental matrix and complete proportions of the evaluated mortars.

Mixture	OPC/FA	OPC (kg m^−3^)	FA (kg m^−3^)	Binder (kg m^−3^)	Sand (kg m^−3^)	Liquid (kg m^−3^)	L/B	A/B	KOHM (g/L)
OPC	100/0	493.44	0.00	493.44	1508.08	395.45	0.80	3.06	–
90/10–4 M	90/10	444.10	49.34	493.44	1508.08	395.45	0.80	3.06	4 (224.4)
90/10–8 M	90/10	444.10	49.34	493.44	1508.08	395.45	0.80	3.06	8 (448.9)
80/20–4 M	80/20	394.75	98.69	493.44	1508.08	395.45	0.80	3.06	4 (224.4)
80/20–8 M	80/20	394.75	98.69	493.44	1508.08	395.45	0.80	3.06	8 (448.9)
70/30–4 M	70/30	345.41	148.03	493.44	1508.08	395.45	0.80	3.06	4 (224.4)
70/30–8 M	70/30	345.41	148.03	493.44	1508.08	395.45	0.80	3.06	8 (448.9)

B=OPC+FA; L/B: liquid-to-binder ratio; A/B: sand-to-binder ratio. In the OPC mixture, *L* corresponds to the mixing water; in the hybrid formulations, it corresponds to the total liquid phase containing the aqueous KOH solution. The KOH dosage is expressed in terms of molarity and mass of KOH per liter of solution.

## Data Availability

The original contributions presented in this study are included in the article. Further inquiries can be directed to the corresponding author.

## Abbreviations

The following abbreviations are used in this manuscript:

ACI	American Concrete Institute
ASTM	ASTM International
EN	European standard
OPC	ordinary Portland cement
FA	fly ash
KOH	potassium hydroxide
M	molar concentration, $\mathrm{mol}\;L^{-1}$
OPC/FA	ordinary Portland cement-to-fly ash ratio
PVC	polyvinyl chloride
FEM	finite element method
FEniCS	finite-element computational platform
Gmsh	finite-element mesh generator
$I_{a}$	specific abrasion index
$I_{a,\mathrm{norm}}$	normalized abrasion index
$I_{m}\left(t\right)$	relative mechanical index at age *t*
$k_{\mathrm{eff}}$	effective microstructural coefficient
$K_{n}$	normalized initial stiffness
$P_{\mathrm{num}}$	numerical reaction force
$\chi_{E}$	stiffness factor
$\chi_{f}\left(t\right)$	strength factor at age *t*
$\chi_{\rho }$	apparent-density factor
$\chi_{w}$	relative wear factor
RMSE	root mean square error
MAE	mean absolute error
